# Alzheimer's Disease Biomarkers Measured by Capillary Blood Collection Versus Venous Plasma and Cerebrospinal Fluid

**DOI:** 10.1002/acn3.70524

**Published:** 2026-10-16

**Authors:** Sara Ho, Ziling Shen, Alexandria Lewis, Megha Patel, Anny Zheng, Gianna Proscino, Thaovy Ho, Jiangxia Wang, Marilyn Albert, Abhay Moghekar

**Affiliations:** ^1^ Johns Hopkins School of Medicine Baltimore Maryland USA; ^2^ Johns Hopkins Bloomberg School of Public Health Baltimore Maryland USA

**Keywords:** Alzheimer's disease, biomarkers, capillary

## Abstract

**Objective:**

Blood biomarkers are increasingly critical for non‐invasive Alzheimer's Disease (AD) diagnostics and research but typically require on‐site processing. Remote point‐of‐care collection methods could enhance biomarker testing in clinical and research settings. We evaluated the safety and accuracy of blood collection via venous blood and two capillary blood collection devices (Tasso+ and TAPII) for measurements of amyloid‐beta 42/40 ratio (Aβ42/40), phosphorylated‐tau‐217 (pTau217), neurofilament‐light (NfL), and glial fibrillary acidic protein (GFAP) from capillary plasma to simultaneously collected venous plasma and cerebrospinal fluid (CSF).

**Methods:**

Patients seen at the Johns Hopkins Center for CSF Disorders (*N* = 192) underwent diagnostic lumbar punctures and venipuncture. Warming packs were held to the skin for 5 min before collecting capillary blood (via TAPII or Tasso+ device) and numeric pain scores. CSF, venous, and capillary plasma were analyzed using Quanterix SIMOA immunoassays. Statistical analysis included Pearson/Spearman correlations, Lasso regression, and quantitative comparisons using Bland–Altman plots.

**Results:**

86 TAPII and 126 Tasso+ plasma samples were collected (77 M, 115 F; mean age 61 ± 18.3 years, range 16–91). Tasso+ devices were 90.3% successful in collecting > 50 μL whole blood, and TAPII devices were 86% successful. Mean pain scores were significantly higher in venous than capillary collections (Venous 2.24/10, TAPII 0.73/10, Tasso+ 0.2/10, *p* < 0.001). Capillary Aβ42/40, NfL, and GFAP strongly correlated with venous plasma analytes (Tasso+: Aβ42/40 *r* = 0.75, NfL *r* = 0.91, GFAP *r* = 0.80, all *p* < 0.001; TAPII: Aβ42/40 *r* = 0.74, NfL *r* = 0.86, GFAP *r* = 0.84, all *p* < 0.001). Tasso+ capillary pTau217 weakly correlated with venous (*r* = 0.23, *p* = 0.04). Capillary NfL and GFAP correlated modestly with CSF NfL and GFAP (TAPII NfL *r* = 0.74, GFAP *r* = 0.6, all *p* < 0.0001; Tasso+ NfL *r* = 0.69, GFAP *r* = 0.59, all *p* < 0.0001). Capillary Aβ42/40 and pTau217 did not significantly correlate with CSF measures. Lasso regression identified age and GFAP as significant predictors of AD across capillary and venous samples (Tasso+ AUC = 0.87, TAPII AUC = 0.75, Venous AUC = 0.83).

**Interpretation:**

Neurodegeneration biomarkers Aβ42/40, NfL, and GFAP in capillary plasma (Tasso+, TAPII) strongly correlated with venous plasma measures. Capillary and venous pTau217 correlated weakly, indicating further assay refinement is needed. Tasso+ collection was less painful and caused less hemolysis compared to TAPII.

## 1 Introduction

Alzheimer's disease (AD) has affected over 50 million people worldwide and is projected to reach 152 million by 2060 [1]. The pathological hallmarks of AD begin to emerge decades before clinical symptoms become apparent. As the disease progresses, tau pathology becomes widespread, marking the transition to the prodromal phase of mild cognitive impairment (MCI), prior to progression to clinical AD dementia [2, 3, 4]. Although testing for AD brain pathology with cerebrospinal fluid (CSF) or positron emission tomography (PET) has been available for over 2 decades, many patients face challenges in accessing the specialized centers performing AD testing and high out‐of‐pocket costs, along with perceived risks [5, 6]. Early diagnosis is crucial for AD management and treatment, as it both assists patients and families in making informed decisions about the future and provides a critical window for clinical intervention [7].

Disease‐modifying treatments (DMTs) are designed to slow the clinical progression of AD via underlying pathophysiological mechanisms involving amyloid or tau [6, 7]. Since Lecanemab, an anti‐amyloid monoclonal antibody, became the first DMT to gain approval from the US Food & Drug Administration (FDA) in 2023, several additional drugs are currently in clinical trial testing [8, 9]. This dual surge in trials seeking participants and patient interest in DMTs created an immediate need for methods to screen eligible patients [10]. Recent advancements in blood biomarker testing for AD offer promising solutions that are more accessible and less invasive for patients [5, 10, 11]. Several high‐sensitivity assays have been developed to reliably detect different p‐tau isoforms in plasma, namely pTau181 and pTau217, which display high performance when discriminating autopsy‐confirmed AD from other neurodegenerative diseases, as well as the β‐amyloid 1–42/1–40 ratio (Aβ42/40) as an enrichment aid for AD trials investigating preclinical and prodromal disease stages [11, 12, 13, 14, 15]. Neurofilament light chain (NfL) is a biomarker of neuroaxonal injury and neurodegeneration, and glial fibrillary acidic protein (GFAP) is a biomarker of astrocyte activation associated with the onset of Aβ deposition; both biomarkers can be reliably measured in CSF and plasma and furthermore, levels demonstrate correlation between sample types [11, 16, 17].

Though blood biomarker tests are expected to meet the growing demand for rapid AD testing, challenges toward widespread implementation, including the requirement for patients to travel, phlebotomists or other trained medical professionals, lab personnel to process specimens, and temporary storage and transport of specimens to a service provider lab [18]. Any disruption to these integral processes could hinder diagnostic AD testing and longitudinal monitoring of DMT clinical trial participants and research study participants [19, 20, 21]. Capillary blood collection devices, developed by several manufacturers in recent years, are designed to collect blood painlessly and non‐invasively from capillaries in the skin. Furthermore, these devices can be used by a caregiver or the patient themselves, if able, from the comfort of their home [18, 22, 23]. To be used for AD diagnostic testing, capillary collection devices must safely and painlessly collect an appropriate amount of sample for biomarker immunoassays [24]. Of the commercially available capillary collection devices, the TAPII and Tasso+ devices were approved by the US FDA in 2019 and 2022, respectively, and were reported to collect 350–600 μL of whole blood on average. Both are described by the manufacturers as single‐use, compact tools used to collect small amounts of capillary whole blood. Respectively, the TAPII is further described as utilizing “gentle vacuum and microneedle technology,” and the Tasso+ is described as a “blood lancing device” [18, 25]. A few studies have reported the use of Tasso+ and TAPII in both clinical and nonclinical settings [26, 27, 28, 29], in assessment of serology [25, 27], hematology [30], and clinical chemistry [29, 31], with good analytical performance and participant acceptance. To our knowledge, there are currently no peer‐reviewed studies evaluating the suitability of capillary plasma collected by TAPII or Tasso+ for diagnostic biomarker testing for Alzheimer's disease.

This study aimed to evaluate and compare the safety, usability, and analytic performance of two capillary blood collection devices for measuring key AD plasma biomarkers—Aβ42/40, pTau217, NfL and GFAP—by comparing capillary plasma measurements to simultaneously collected venous plasma and CSF measurements.

## 2 Methods

### 2.1 Research Participants

CSF was obtained from 192 patients examined in the Johns Hopkins Center for CSF disorders between October 2021 and April 2024 who provided consent for biospecimen banking for research. This group included patients with a range of neurologic disorders [e.g., normal pressure hydrocephalus (NPH) and AD] and patients with no history of cognitive disorder who were referred for lumbar puncture (LP) for assessment of headache with normal opening pressures and CSF. Patients diagnosed with idiopathic NPH met standard guidelines [32]. Patients diagnosed with AD satisfied National Institute on Aging–Alzheimer's Association criteria based on clinical, cognitive, and neuroimaging assessments [33]. An “Other” diagnostic group was defined to include participants with non‐Alzheimer's neurological conditions, including non‐AD neurodegenerative disorders and idiopathic intracranial hypertension. This group was included to reflect real‐world clinical heterogeneity and to evaluate biomarker performance across a range of neurological conditions. All participants underwent clinical evaluation including neurological examination, neuroimaging, and cognitive assessment (MoCA, CDR), with additional testing performed as clinically indicated.

Standard protocol approvals, registrations, and patient consents.

The Institutional Review Board of Johns Hopkins School of Medicine approved this study. All participants provided written informed consent before participation.

### 2.2 CSF Collection

CSF was collected via diagnostic large volume lumbar puncture (LP). Under sterile conditions, patients each had a spinal needle or catheter placed between the L3/L4 or L4/L5 intervertebral space. Fresh CSF was collected in 10 mL polypropylene tubes and transported to the lab on ice.

### 2.3 Venous and Capillary Blood Collection

Patients underwent venipuncture on one arm and capillary collection on the other using either TAPII or Tasso+ device, both containing EDTA as an anticoagulant. All venous whole blood samples were collected in tubes containing EDTA (Vacutainer, BD). Prior to device placement, thorough skin cleaning on the upper arm site was completed before a warming pack was placed for 5 min as per manufacturer's protocol (see Figure S1). TAPII or Tasso+ devices were placed, and collection of blood occurred over 5 min. Patients were asked to provide Numeric Rating Scale (NRS) scores (range: 0–10) for pain experienced during venous and capillary collections. Samples were inverted immediately and transported together to the lab.

### 2.4 Neurocognitive Testing

The Montreal Cognitive Assessment (MoCA) test and Clinical Dementia Rating Sum of Boxes (CDR‐SOB) were both used to assess cognitive performance.

### 2.5 Sample Processing and Biochemical Analysis

Venous and capillary whole blood samples were centrifuged (2000 *g*, 4°C) for 15 min before plasma was extracted and centrifuged a second time to remove cellular debris. CSF was centrifuged (2000 *g*, 4°C) for 15 min, transferred to a new tube, and centrifuged once more. Samples were frozen at −80°C within 90 min of collection. Before analysis, CSF, venous plasma, and capillary plasma were thawed for 1 h on ice and vortexed for 10 s. Plasma samples were centrifuged for 10,000 *g* for 5 min.

The levels of Aβ40, Aβ42, pTau217, NfL, and GFAP in CSF, venous plasma, and capillary plasma were measured in singlicate using a Simoa HD‐X analyzer (Quanterix) and commercially available kits (Neurology 4‐Plex E assay kit for GFAP, NfL, Aβ40, and Aβ42; ALZpath pTau217 assay for pTau217). Hemolysis of capillary plasma samples was determined via Roche Combur‐Test erythrocyte/hemoglobin strips [34]. A native plasma control sample was monitored in addition to the low and high kit controls across all plates. Inter‐assay coefficients of variation were < 10% for Aβ42, < 8% for Aβ40, < 9% for NfL, < 12% for GFAP and < 7% for pTau217. Intra‐assay coefficients on variation were < 7% for Aβ42, < 4% for Aβ40, < 6% for NfL, < 8% for GFAP and < 5% for pTau217.

### 2.6 Statistical Analysis

All participants with available biomarker and diagnostic data were included in the analysis, and distributional assumptions were assessed using Shapiro–Wilk tests and *Q*–*Q* plots for all tests to determine whether parametric or non‐parametric procedures were appropriate. When a non‐parametric procedure (Spearman's correlation test, Wilcoxon rank‐sum test, etc.) without distributional assumption such as normality was applied, no log transformation was needed. When a parametric procedure (Pearson's correlation test, *t*‐test, etc.) with normality assumption, log transformation was applied to the data before the analysis was conducted to be consistent with how the biomarkers were analyzed in other studies. Exploratory data analysis was performed to examine and eliminate outliers with boxplots. Statistical analyses used in this study included Pearson's and/or Spearman's correlation to evaluate the relationships between biomarker concentrations across sample types (capillary, venous, and CSF). Log transformation was applied to Pearson correlation when necessary with both measurements being normally distributed according to Shapiro–Wilk test. Spearman's correlation was used when neither measurement is normally distributed. Wilcoxon rank‐sum tests were used to compare continuous outcomes between diagnostic groups, assess differences in biomarker levels, and pain or hemolysis scores across collection methods. For multiple hypothesis tests with the same research question, Bonferroni's correction was used to avoid inflating Type I error. Bland–Altman analysis of agreement was performed to assess the level of agreement between TAPII/TASSO+ and venous with 95% limits of agreement. Passing–Bablok regression was additionally performed to evaluate constant and proportional bias between capillary and venous biomarker measurements. Intercepts with 95% confidence intervals including zero were interpreted as absence of constant bias, while slopes with confidence intervals including one were interpreted as absence of proportional bias. Linearity assumptions were assessed using the cumulative sum (CUSUM) test. Intraclass Correlation Coefficient (ICC) was also calculated to quantify the similarity between two measurements. Age‐adjusted logistic regression models were additionally constructed for each biomarker separately to compare the diagnostic performance of venous and capillary plasma measurements for classification of Alzheimer's disease. Area under the receiver operating characteristic curve (AUC) values with 95% confidence intervals were calculated for each sample type, and AUCs were compared across venous, TAPII, and Tasso+ measurements. Lasso logistic regression with a binary outcome of diagnosis of Alzheimer's disease was selected to account for small sample size of people with positive diagnosis (*n* = 25) and to reduce risk of overfitting. Multiple models were constructed to identify the most informative combination of demographic and biomarker variables for predicting a clinical diagnosis of Alzheimer's disease. All analyses were performed in R with a significance level of 0.05.

## 3 Results

### 3.1 Participant Characteristics

A total of 192 participants were included in the study with mean age 61 years ±18.3 (range 16–91 years). 77 participants were male and 115 were female. Participants were divided by age and clinical diagnosis into patient groups: Normal Pressure Hydrocephalus, Alzheimer's Disease, Vascular Dementia (VaD), Older Normal (ON) for cognitively normal patients above 60 years of age, and Younger Normal (YN) for cognitively normal patients below 60 years of age. A number of patients met diagnostic criteria for separate neurological conditions, namely dementia with Lewy bodies (DLB), frontotemporal dementia (FTD), Parkinson's Disease (PD), progressive supranuclear palsy (PSP), limbic‐predominant age‐related TDP‐43 encephalopathy (LATE), and idiopathic intracranial hypertension (IIH), and were included in the “Other” patient group as described in Methods. 81.77% of participants were White, 14.06% were Black, 1.04% were Hispanic, 1.56% were Asian, and 1.56% were Other (Table 1). See Figure 1 for full study design.

**TABLE 1:** Participant demographics and clinical characteristics.

	Overall	YN	ON	NPH	AD	VaD	Other
Number of participants	192	25	11	42	25	28	61
Gender, *n* (%)							
Men	77 (40.1%)	4 (16%)	3 (27.3%)	21 (50%)	15 (60%)	21 (75%)	13 (21.3%)
Women	115 (60%)	21 (84%)	8 (72.7%)	21 (50%)	10 (40%)	7 (25%)	48 (78.7%)
Age, mean (SD)	61.5 (18.4)	47.9 (17.9)	75.6 (5.1)	41.7 (9.1)	68.8 (6)	72.7 (8.8)	76.3 (5.4)
Race/Ethnicity, *n* (%)							
White	157 (81.8%)	18 (72%)	11 (100%)	38 (90.5%)	24 (96%)	26 (92.9%)	40 (65.6%)
Black or African American	27 (14%)	5 (20%)	0 (0%)	1 (2.4%)	1 (4%)	0 (0%)	20 (32.8%)
Hispanic or Latino	2 (1%)	1 (4%)	0 (0%)	1 (2.4%)	0 (0%)	0 (0%)	0 (0%)
Asian	3 (1.6%)	1 (4%)	0 (0%)	1 (2.4%)	0 (0%)	1 (3.6%)	0 (0%)
Other	3 (1.6%)	0 (0%)	0 (0%)	1 (2.4%)	0 (0%)	1 (3.6%)	1 (1.6%)
BMI, mean (SD)	31.1 (7.7)	34.5 (8.6)	27.5 (4.6)	36.6 (8.1)	30.2 (5.2)	28.7 (5)	25.8 (4.7)
CDR‐SOB, mean (SD)	1.7 (2.3)	1.1 (2.8)	1.8 (2)	0.6 (0.9)	0.9 (2.2)	2.3 (2)	3.3 (2.2)
MoCA, mean (SD)	23.7 (4.1)	24.1 (4.1)	22.1 (3)	26.6 (2.1)	27.6 (1.6)	23.1 (2.8)	21.2 (5.9)

Abbreviations: AD, Alzheimer's disease; NPH, normal pressure hydrocephalus; ON, old normal; VaD, vascular dementia; YN, young normal.

**FIGURE 1:**
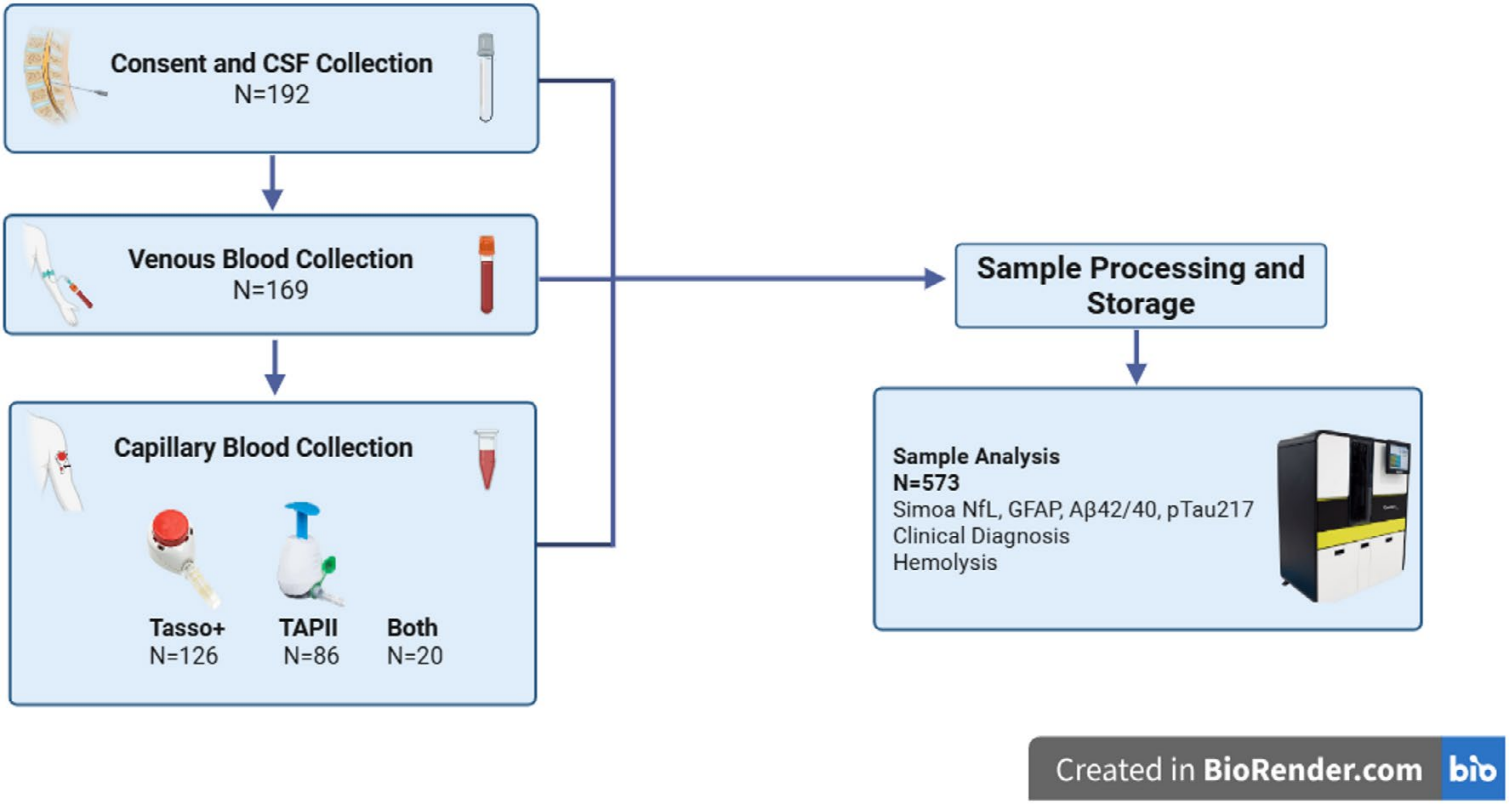
Overview of participant enrollment and biospecimen collection. Following informed consent, cerebrospinal fluid (CSF) was collected from 192 participants where available, and venous blood samples were obtained by standard phlebotomy in 169 participants. Capillary blood was collected using two FDA‐cleared devices, Tasso+ (*n* = 126) and TAPII (*n* = 86), with 20 participants providing samples using both devices. Numbers indicate samples collected for each biospecimen type.

### 3.2 Sample Characteristics

191 CSF, 169 venous plasma, 86 TAPII, and 126 Tasso+ capillary plasma samples were collected throughout the study from 192 participants (20 patients had both Tasso+ and TAPII collections) (Table S1). This was a convenience sample and patients were not randomized to TAPII or Tasso+ as devices were made available to us sequentially by the manufacturers. Venous plasma could not be obtained by traditional phlebotomy in 24 participants due to technical challenges. The mean volume for Tasso+ samples was 294.1 μL ±127.2 and 266.94 μL ±18.71 for TAPII. Tasso+ devices were 90.3% successful in collecting > 50 μL whole blood, and TAPII devices were 86% successful. Differences between mean volumes and success rates were not statistically significant (*p* = 0.108, *p* = 0.258 respectively). The mean biomarker values and standard deviation of each patient group can be found in Table S2.

### 3.3 Correlation of Capillary and Venous Biomarker Measurements

Capillary NfL, GFAP, and Aβ_42/40_ from both TAPII and Tasso+ strongly correlated with venous plasma NfL, GFAP, and Aβ42/40 (TAPII NfL *r* = 0.86, *p* < 0.001, GFAP *r* = 0.84, *p* < 0.001, Aβ42/40 *r* = 0.74, *p* < 0.001; Tasso+ NfL *r* = 0.91, *p* < 0.001, GFAP *r* = 0.8, *p* < 0.001, Aβ42/40 *r* = 0.75, *p* < 0.001). Tasso+ capillary plasma pTau217 only weakly correlated with venous pTau217 (*r* = 0.23, *p* = 0.04) and TAPII pTau217 did not significantly correlate with venous pTau217 (*p* = 0.12) (Figures 2 and 3).

**FIGURE 2:**
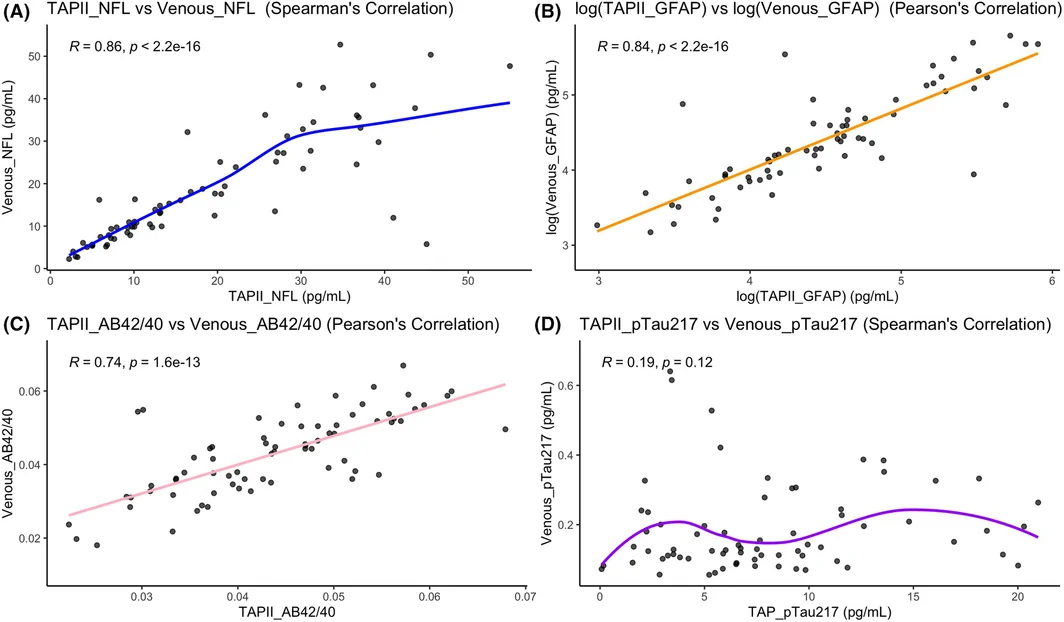
Correlation between venous plasma levels and NfL (A), GFAP (B), Aβ42/40 (C), and pTau217 (D) levels from TAPII capillary plasma. Scatterplots show correlations between matched venous plasma concentrations and biomarker levels measured from TAPII‐derived capillary plasma for (A) neurofilament light chain (NfL), (B) glial fibrillary acidic protein (GFAP), (C) amyloid‐β42/40 (Aβ42/40), and (D) phosphorylated tau 217 (pTau217). Spearman rank correlation was used for NfL and pTau217, and Pearson correlation was used for GFAP (log‐transformed values) and Aβ42/40, as indicated in each panel. Each point represents paired samples from the same participant. Correlation coefficients (R) and *p* values are shown within each panel.

**FIGURE 3:**
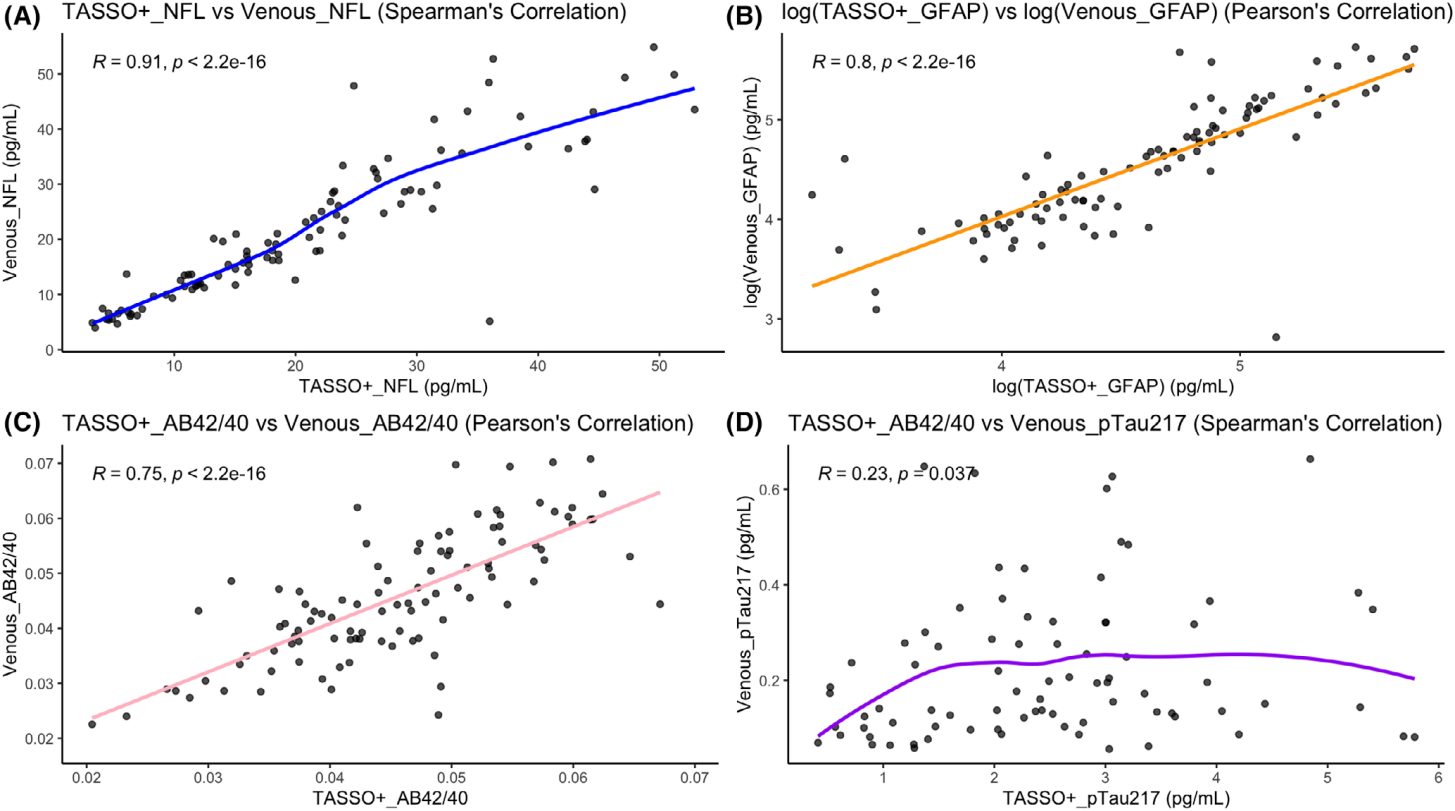
Correlation between venous plasma levels and NfL (A), GFAP (B), Aβ42/40 (C), and pTau217 (D) levels from Tasso+ capillary plasma. Scatterplots show correlations between matched venous plasma concentrations and biomarker levels measured from Tasso+‐derived capillary plasma for (A) neurofilament light chain (NfL), (B) glial fibrillary acidic protein (GFAP), (C) amyloid‐β42/40 (Aβ42/40), and (D) phosphorylated tau 217 (pTau217). Spearman rank correlation was used for NfL and pTau217, and Pearson correlation was used for GFAP (log‐transformed values) and Aβ42/40, as indicated in each panel. Each point represents paired capillary and venous plasma samples from the same participant. Correlation coefficients (R) and *p* values are shown within each panel.

### 3.4 Pairwise Comparison of Analytes in Venous and Capillary Plasma Samples

Overall, no significant differences were observed between TAPII, Tasso+, and venous Aβ42/40, NFL, and GFAP measurements after pairwise comparisons between sample types. In contrast, pTau217 measurements were significantly different when comparing TAPII, Tasso+, and venous samples (all *p* < 0.0001) as seen in Figure S2.

### 3.5 Bland–Altman Analysis of Bias Among Analyte Concentrations

Bland–Altman plots revealed varying levels of agreement between capillary and venous samples across biomarkers. For AB42/40, both devices showed minimal bias and tight agreement across the range of values. NFL and GFAP showed wider limits of agreement, particularly for GFAP, which demonstrated substantial variability and multiple outliers for both TAPII and Tasso+. Analysis of pTau217 demonstrated a consistent positive bias for both TAPII and Tasso+ versus venous samples with mean biases of approximately 7 pg/mL for TAPII and 3 pg/mL for Tasso+. This overestimation became more pronounced at higher pTau217 concentrations, although the 95% limits of agreement remained within clinically acceptable bounds (Figures 4 and 5). These findings suggest excellent agreement for AB42/40, systematic bias for pTau217, and less consistent agreement for NFL and GFAP. Intraclass correlation coefficients and other Bland–Altman metrics were included in Table S3.

**FIGURE 4:**
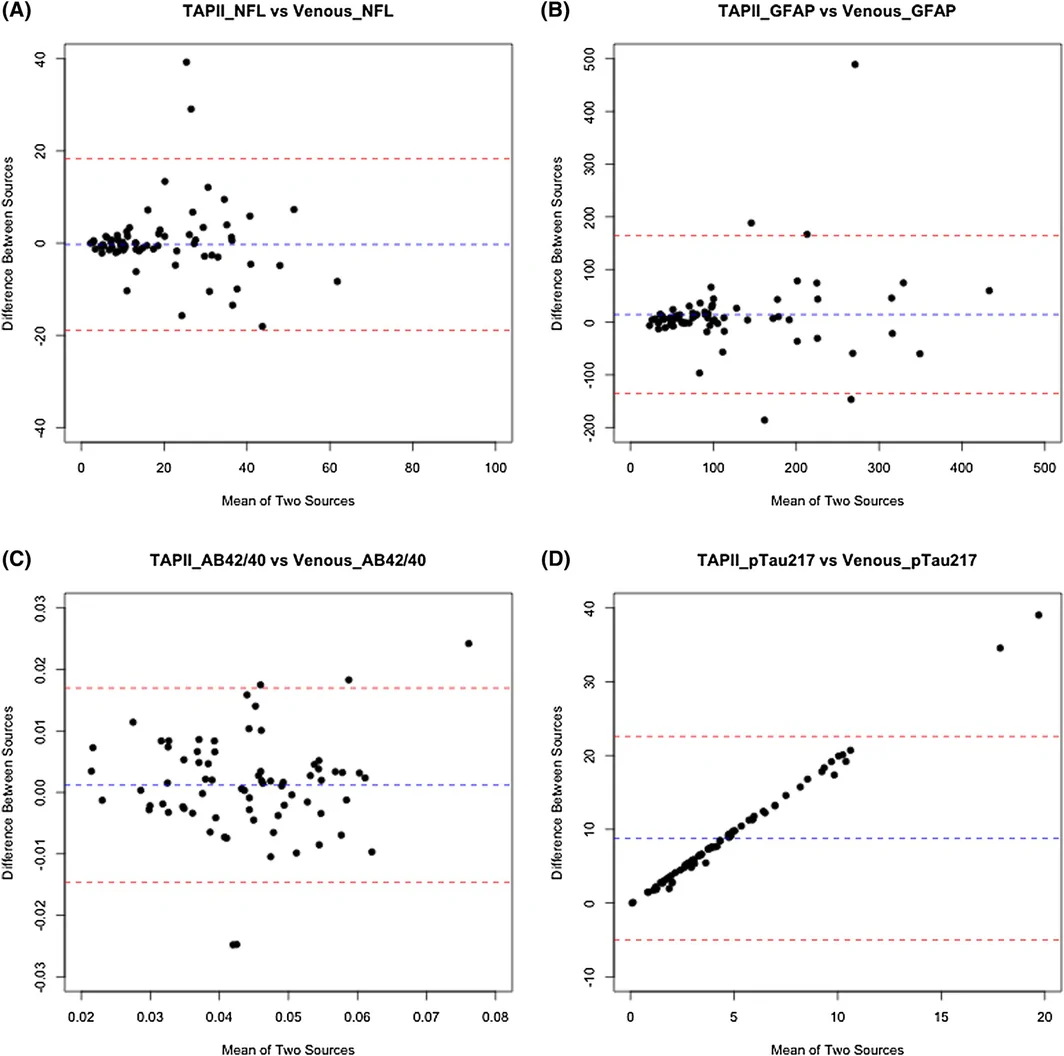
Bland Altman analysis of TAPII and venous NfL (A), GFAP (B), Aβ42/40 (C), and pTau217 (D) levels. Bland–Altman plots show agreement between TAPII‐derived capillary plasma and matched venous plasma measurements for (A) neurofilament light chain (NfL), (B) glial fibrillary acidic protein (GFAP), (C) amyloid‐β42/40 (Aβ42/40), and (D) phosphorylated tau 217 (pTau217). The *x*‐axis represents the mean of capillary and venous measurements, and the *y*‐axis represents the difference between the two methods. The dashed line indicates the mean difference (bias), and the outer dashed lines indicate the limits of agreement (±1.96 standard deviations). Each point represents paired samples from the same participant.

**FIGURE 5:**
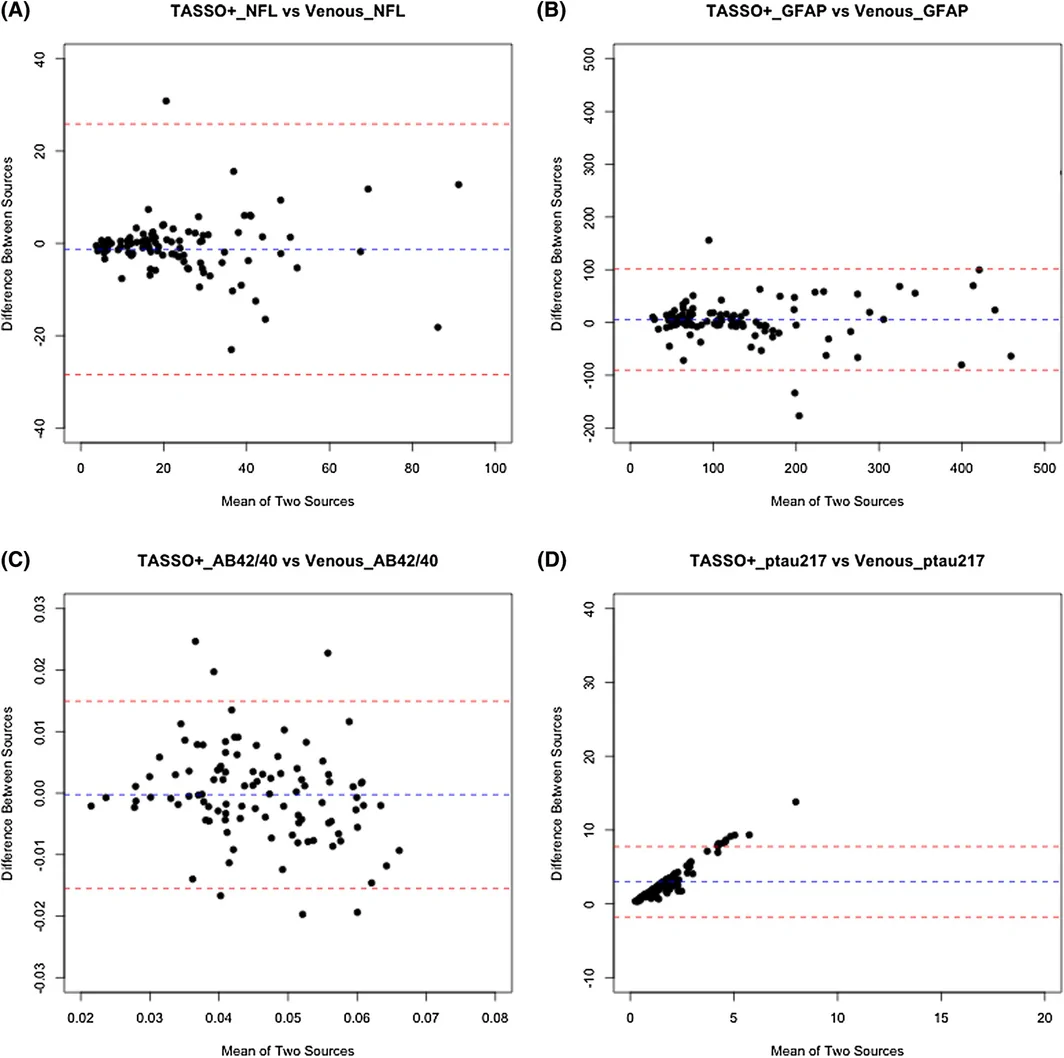
Bland Altman analysis of Tasso+ and venous NfL (A), GFAP (B), Aβ42/40 (C), and pTau217 (D) levels. Bland–Altman plots show agreement between Tasso+‐derived capillary plasma and matched venous plasma measurements for (A) neurofilament light chain (NfL), (B) glial fibrillary acidic protein (GFAP), (C) amyloid‐β42/40 (Aβ42/40), and (D) phosphorylated tau 217 (pTau217). The *x*‐axis represents the mean of capillary and venous measurements, and the *y*‐axis represents the difference between the two methods. The dashed line indicates the mean difference (bias), and the outer dashed lines indicate the limits of agreement (±1.96 standard deviations). Each point represents paired samples from the same participant.

### 3.6 Passing–Bablok Regression Analysis of Capillary and Venous Biomarker Agreement

Passing–Bablok regression analysis demonstrated strong agreement between capillary and venous measurements for NfL, GFAP, and Aβ42/40 across both TAPII and Tasso+ devices. For these biomarkers, most intercept confidence intervals included zero and slope confidence intervals included one, indicating minimal evidence of constant or proportional bias. In contrast, pTau217 demonstrated substantial proportional bias for both TAPII and Tasso+ devices, with slope estimates markedly deviating from one, consistent with the systematic overestimation observed in Bland–Altman analysis. CUSUM tests supported the assumption of linearity for all biomarker comparisons (all *p* > 0.15). Full Passing–Bablok regression statistics are shown in Table [Link], [Link].

### 3.7 CSF Biomarker Measurements Versus Venous and Capillary Plasma

Venous Aβ42/40 did not significantly correlate with CSF Aβ42/40 (*p* = 0.169). Venous NfL, GFAP, and pTau217 all significantly correlated with CSF analytes (NfL *r* = 0.73, *p* < 0.001, GFAP *r* = 0.59, *p* < 0.001, pTau217 *r* = 0.33, *p* < 0.001). Capillary NfL and GFAP correlated modestly with CSF NfL and GFAP (TAPII NfL *r* = 0.74, *p* < 0.0001, GFAP *r* = 0.6, *p* < 0.001; Tasso+ NfL *r* = 0.69, *p* < 0.001, GFAP *r* = 0.59, *p* < 0.001) whereas capillary Aβ42/40 and pTau217 did not significantly correlate with CSF measures (Figures S3 and S4).

### 3.8 Age‐Adjusted Diagnostic Performance of Individual Biomarkers

To further compare the diagnostic performance of individual biomarkers across sample types, age‐adjusted logistic regression models were constructed separately for venous, TAPII, and Tasso+ measurements. Across all sample types, NfL, GFAP, Aβ42/40, and pTau217 demonstrated comparable discrimination of Alzheimer's disease status, with no statistically significant differences in AUC observed between venous and capillary measurements (all *p* > 0.05). Venous pTau217 demonstrated the highest individual biomarker performance (AUC 0.88, 95% CI 0.82–0.94), while GFAP and Aβ42/40 demonstrated similar diagnostic performance across capillary and venous sample types. Full age‐adjusted AUC values are presented in Table S5. Evaluation of biomarker performance for amyloid assessment in AD patients.

Lasso regression was used to identify which biomarkers and demographic variables most strongly predicted Alzheimer's disease status across all sample types. Multiple imputation using predictive mean matching was performed before fitting the model. 10‐fold cross validation was used to select the optimal lambda value. Among the candidate predictors included in the model, age and plasma GFAP levels consistently emerged in all sample types as significant contributors to classification performance. Specifically, models incorporating these variables yielded area under the curve (AUC) values of 0.75 (0.70, 0.85) for TAPII‐derived capillary plasma, 0.87 (0.79, 0.90) for Tasso+‐derived capillary plasma, and 0.83 (0.73, 0.88) for venous plasma. For the TAPII model, Aβ42/40 was additionally selected along with age and GFAP. See Table S6 for full Lasso regression metrics.

### 3.9 Pain and Hemolysis Scores of Capillary and Venous Samples

Mean NRS scores of pain were significantly lower across Tasso+ collections when compared to traditional venous venipuncture and TAPII capillary collection (Mean NRS TAPII 0.73/10, Tasso+ 0.2/10, Venous 2.24/10, TAPII vs. Tasso+ *p* < 0.001, venous vs. TAPII *p* < 0.001, venous vs. Tasso+ *p* < 0.001). Hemolysis scores (TAPII 2.08/4, Tasso+ 1.37/4) were significantly higher in TAPII collections (*p* < 0.001) (Figure 6). Hemolysis did not significantly alter the relationship between capillary and venous biomarker measurements. Although minor variation in correlation strength was observed across hemolysis levels, the association between capillary and venous levels remained strong and statistically significant overall.

**FIGURE 6:**
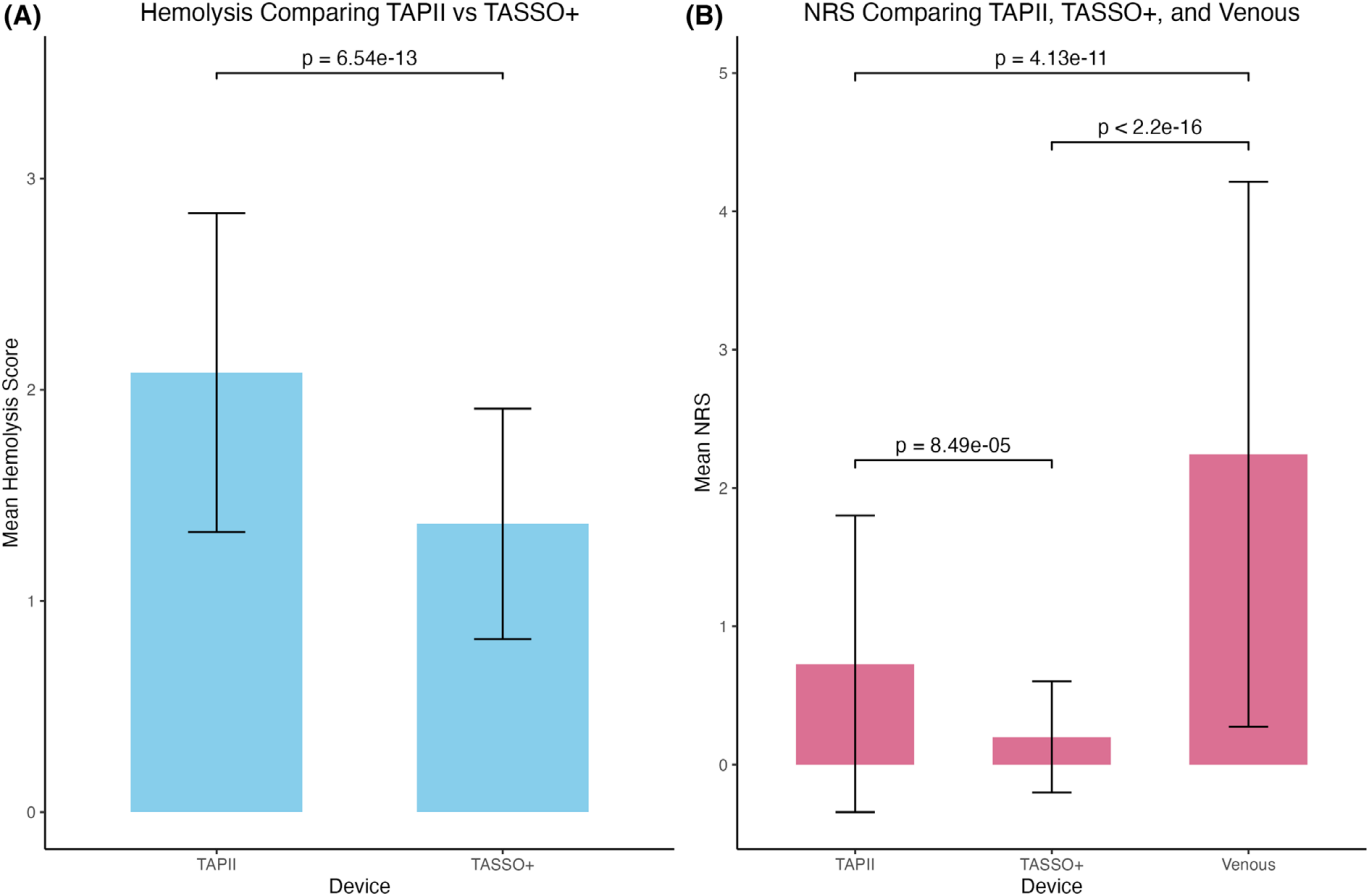
Comparison of hemolysis scores of Tasso+ and TAPII samples (A), comparison of NRS scores of Tasso+ and TAPII patient collections of samples (B). Data are mean ± SD. Bar plots compare (A) hemolysis scores between TAPII and Tasso+ capillary blood collection devices and (B) participant‐reported pain measured using the Numeric Rating Scale (NRS) across TAPII, Tasso+, and venous blood collection methods. Bars represent mean values, with error bars indicating standard deviation. Pairwise statistical comparisons are indicated above brackets with corresponding *p* values. Hemolysis was assessed as a measure of sample quality, and NRS scores were collected to evaluate participant‐reported pain associated with each collection method.

### 3.10 Correlations Between Capillary Biomarker Measurements and Neurocognitive Measures

With regards to MoCA and CDR‐SOB scores, there were no observed significant correlations with any capillary biomarker measurements (Table S8).

## 4 Discussion

This study evaluated the utility of capillary blood collected via TAPII and Tasso+ devices for measuring plasma biomarkers associated with neurodegenerative disease. Among 192 participants representing a range of clinical diagnoses, capillary concentrations of Aβ42/40, NfL, and GFAP demonstrated strong correlation and minimal bias relative to venous plasma. In contrast, pTau217 showed consistent overestimation in capillary samples and limited correlation with venous values, particularly in TAPII collections. Lasso regression identified age and GFAP as the most consistent predictors of Alzheimer's disease across all sample types, with the highest classification accuracy observed in Tasso+‐derived samples. Pain and hemolysis scores indicated that Tasso+ was better tolerated and associated with lower sample interference compared to both TAPII and venous collection. These results support the feasibility of capillary collection for certain plasma biomarkers, while underscoring the need for further refinement in pTau217 measurement. Capillary plasma concentrations of amyloid‐beta 42/40 ratio (Aβ42/40), neurofilament‐light (NfL), and glial fibrillary acidic protein (GFAP) correlated strongly with their venous‐plasma counterparts, indicating that immunoassays originally optimized for venous samples yield reliable results for these analytes when applied to capillary specimens. A previous study by Huber et al., which measured capillary AD biomarkers collected using dried blood spots, also measured strong correlations between capillary and venous NfL and GFAP but only found a weak, nonsignificant correlation between capillary and venous Aβ42/40 [35]. When comparing CSF to venous plasma markers, we observed significant correlations in NfL, GFAP, and pTau217 measurements, in concordance with previous CSF‐plasma AD biomarker studies; however, when measuring correlations between CSF and capillary plasma markers, only NfL and GFAP demonstrated significant correlations [36, 37, 38].

Surprisingly, in our data pTau217 did not demonstrate any significant correlation between capillary and venous measurements for either device, which contradicts findings by Huber et al. [35]. Additionally, over 95% of capillary pTau217 concentrations measured by TAPII or Tasso+ across all patient groups, even controls, all exceeded the preliminary cutoff of 0.42 pg/mL used in established literature to indicate significant brain amyloid pathology supporting a biomarker verified diagnosis of AD [39]. In comparison, for venous plasma samples the average pTau217 measurement only exceeded the 0.42 pg/mL cutoff in the AD group and the “Other” patient group, primarily comprised of mixed neurodegenerative conditions (Table S2, Figures S5 and S6). This significant difference between pTau217 levels across sample types represents a nearly 30‐fold difference between TAPII and venous measurements, a 10‐fold difference between Tasso+ and venous measurements, and a 3‐fold difference between TAPII and Tasso+ pTau217 measurements, presenting a crucial obstacle to utilizing blood biomarker tests developed using venous plasma for capillary plasma samples without extensive further optimization. Furthermore, Bland–Altman and Passing–Bablok analyses demonstrated that for both TAPII and Tasso+ devices, as the mean values of capillary pTau217 increased, the difference between capillary and venous measurements also increased, consistent with proportional bias between sample types (Figures 4 and 5; Table S4). Published studies correlating capillary AD biomarkers to venous plasma markers to compare our results to are still lacking; however, several studies have established that phosphorylated tau species, including pTau217, as well as α‐synuclein aggregates, exist in peripheral nerve terminals of the skin, presenting a plausible hypothesis that capillary collection devices may release peripheral pTau217 into the whole blood sample collected, thereby increasing the overall concentration [40, 41, 42]. Also, matrix effects unique to the pTau217 assay may be involved as all the other analytes are run on a different assay kit. In our study, differences in the dimensions and number of needles utilized in the TAPII and Tasso+ devices may explain variation in pTau217 concentration as well as hemolysis even among different capillary collection devices; as reported by the manufacturers and by previous studies utilizing these devices, the TAPII uses an array of 16 microneedles, each 500 μm in length, and the Tasso+ uses a single lancet, 5 mm in length [43, 44, 45, 46]. See Figure S7 for images of TAPII and Tasso+ inner mechanisms. Hemolysis, which was significantly higher in capillary samples compared to venous, had minimal impact on the correlation between capillary and venous measurements of Aβ42/40, NfL, and GFAP, with all three demonstrating robust relationships across hemolysis levels. In contrast, pTau217 exhibited weaker overall correlation and a trend toward greater sensitivity to hemolysis (Table S7). Since the full effect of preanalytical variables such as hemolysis has yet to be determined, capillary blood biomarker tests will need to be thoroughly evaluated as CSF and venous plasma biomarker assays have been over the past decade, which may necessitate separate cutoffs and guidelines for these factors in different plasma sample types to account for this measurement gap.

Using Lasso‐regularized logistic regression to evaluate all combinations of Alzheimer's disease (AD) biomarkers and demographic variables across sample types, we found that age and glial fibrillary acidic protein (GFAP) consistently provided the highest discriminative power for distinguishing AD from non‐AD participants. Age remains the dominant demographic risk factor for AD, while GFAP has recently gained recognition as an early marker of astrocytic activation in response to amyloid pathology [2, 17]. Importantly, the high AUC of 0.87 in the Tasso+ model, despite the absence of other analytes, suggests this collection modality offers analytically viable and minimally invasive access to key AD risk information. These results support future use of capillary GFAP, particularly in combination with age or APOE genotype, as a scalable screening tool for AD in primary care or decentralized research settings and suggest that GFAP is a biologically relevant capillary biomarker. While NFL is elevated in AD, it is also elevated in many of the other neurodegenerative diagnoses present in our study, shown in the weak discriminatory power between AD and non‐AD participants [47]. Additionally, our finding of decreased predictive utility for capillary Aβ42/40 concurs with several previous studies citing increased diagnostic power of the plasma amyloid ratio in preclinical and MCI stages of AD, whereas our AD cohort primarily comprised clinical AD dementia [38, 48, 49, 50]. Although plasma pTau217 has consistently outperformed other phosphorylated tau isoforms in distinguishing Alzheimer's disease from other dementias and in predicting PET positivity, its exclusion from our Lasso models likely reflects limitations in capillary pTau217 measurement. These limitations may be related to assay performance, platform‐specific variability, and pre‐analytical factors such as hemolysis or processing delays, all of which can disproportionately affect tau quantification [51, 52, 53, 54]. Unexpectedly, even with just age and GFAP as predictive variables, the AUC value generated by the Tasso+ model exceeded TAPII and venous models in our study and fell close to AUC values generated by plasma AD biomarker testing in larger studies [50].

Our findings highlight the logistical advantages of capillary collection and minimal loss in diagnostic signal for certain biomarkers compared to venous plasma, both crucial factors for remote or decentralized trial enrollment, primary care screening, or longitudinal, low‐burden monitoring workflows not just in Alzheimer's diagnosis, treatment, and research, but across the entire medical field. A key strength is that we evaluated two distinct capillary blood collection devices, TAPII and Tasso+, in parallel with venous and CSF sampling, allowing direct comparison of biomarker performance across matrices and platforms within the same individuals. Unlike prior studies limited to amyloid‐confirmed cohorts, our sample included a clinically diverse population spanning multiple diagnostic categories and age groups, enhancing the real‐world generalizability of our findings [55, 56]. Lastly, in order to determine the safety and acceptability of these devices by elderly patients, pain was measured through NRS after venous venipuncture and capillary collection; since pain scores were significantly lower after capillary collections, particularly Tasso+, patients appear to prefer them over traditional blood draws. Other studies using capillary collection devices have reported similar findings with regard to pain, indicating that lower pain associated with capillary collection may improve patient compliance for blood draws [19, 22]. Furthermore, mean volumes collected by TAPII and Tasso+ devices differed slightly, but both devices yielded sufficient blood for multi‐biomarker assays in > 85% of collections and comparable success rates compared to traditional venipuncture.

Our study has several limitations. The cohort included heterogeneous underlying diagnoses and was not randomized between capillary collection devices, which may introduce bias but reflects real‐world clinical conditions [57, 58, 59]. Variability in capillary collection volume may alter the EDTA‐to‐plasma ratio; however, strong agreement with venous measurements suggests minimal impact under the conditions tested. Additionally, the study was conducted in a supervised clinical setting, and findings may differ in unsupervised or at‐home collection environments.

The number of clinically diagnosed AD cases was relatively small (*n* = 25), which may have limited statistical power and contributed to variability in classification performance. Interpretation of the weak correlation between capillary and venous pTau217 is further limited by the lack of established reference ranges or validation studies in capillary plasma, making it difficult to distinguish between biological, matrix‐related, or assay‐specific effects [60].

Future studies should validate these findings in larger, biomarker‐confirmed cohorts and further investigate pTau‐based ratios in capillary plasma [58, 61, 62]. Additional work is also needed to evaluate at‐home collection feasibility and to assess longitudinal biomarker changes over time.

## 5 Conclusion

Although these findings overall support the feasibility of capillary biomarker testing and use of capillary collection devices in biomarker testing in decentralized AD clinical trials and studies, a clear need exists for specific guidelines and assay optimization for capillary biomarkers due to significant technical or biological differences in pTau217 between TAPII, Tasso+ and venous plasma samples. Future studies would support a vision in which patients or caregivers could provide a small capillary sample at home or a clinic, and clinicians could triage them for confirmatory CSF or PET testing based on algorithmic risk.

## Funding

This work was supported by the National Institute on Aging (Grants P30AG066507 and U19AG033655) and philanthropic contributions by the Lantry Family Foundation.

## Conflicts of Interest

Sara Ho, Ziling Shen, Alexandria Lewis, Megha Patel, Anny Zheng, Gianna Proscino, Thaovy Ho, Jiangxia Wang, and Marilyn Albert declare no conflicts of interest. Abhay Moghekar reports research support from Fujirebio Diagnostics Inc.

## Supporting information


**Figure S1:** Tasso+ manufacturer instructions for use (IFU). Manufacturer‐provided instructions for use for the Tasso+ capillary blood collection device.

**Figure S2:** Mean pTau217 concentrations across sample types. Figure illustrating mean phosphorylated tau 217 (pTau217) concentrations and standard deviations for TAPII, Tasso+, and venous plasma samples, with pairwise comparisons between sample types.

**Figure S3:** Correlations between TAPII‐derived plasma and CSF biomarkers. Scatterplots showing correlations between TAPII‐derived capillary plasma and cerebrospinal fluid (CSF) measurements for (A) neurofilament light chain (NfL), (B) glial fibrillary acidic protein (GFAP), (C) amyloid‐β42/40 (Aβ42/40), and (D) phosphorylated tau 217 (pTau217).

**Figure S4:** Correlations between Tasso+‐derived plasma and CSF biomarkers. Scatterplots showing correlations between Tasso+‐derived capillary plasma and cerebrospinal fluid (CSF) measurements for (A) neurofilament light chain (NfL), (B) glial fibrillary acidic protein (GFAP), (C) amyloid‐β42/40 (Aβ42/40), and (D) phosphorylated tau 217 (pTau217).

**Figure S5:** Comparison of TAPII and venous plasma biomarkers by diagnostic group. Figure comparing average concentrations and standard deviations of (A) neurofilament light chain (NfL), (B) glial fibrillary acidic protein (GFAP), (C) amyloid‐β42/40 (Aβ42/40), and (D) phosphorylated tau 217 (pTau217) between TAPII‐derived capillary plasma and venous plasma samples stratified by cognitively normal (ON) and Alzheimer's disease (AD) groups.

**Figure S6:** Comparison of Tasso+ and venous plasma biomarkers by diagnostic group. Figure comparing average concentrations and standard deviations of (A) neurofilament light chain (NfL), (B) glial fibrillary acidic protein (GFAP), (C) amyloid‐β42/40 (Aβ42/40), and (D) phosphorylated tau 217 (pTau217) between Tasso+‐derived capillary plasma and venous plasma samples stratified by cognitively normal (ON) and Alzheimer's disease (AD) groups.

**Figure S7:** Internal sampling components of capillary blood collection devices. Representative images of the internal sampling components of the (A) Tasso+ inner lancet and (B) TAPII inner microneedle ring.

**Table S1:** Sample characteristics by collection method. Table summarizing participant sample characteristics stratified by blood collection method.

**Table S2:** Mean concentrations and standard deviations of biomarkers across sample types. Table reporting mean concentrations and standard deviations of neurofilament light chain (NfL), glial fibrillary acidic protein (GFAP), amyloid‐β42/40 (Aβ42/40), and phosphorylated tau 217 (pTau217) in TAPII, Tasso+, venous plasma, and cerebrospinal fluid (CSF) samples.

**Table S3:** Intraclass correlation coefficients and Bland–Altman agreement metrics. Table summarizing intraclass correlation coefficients and Bland–Altman agreement metrics comparing capillary and venous plasma biomarker measurements.

**Table S4:** Passing–Bablok regression statistics for capillary and venous biomarker agreement. Table summarizing Passing–Bablok regression analyses comparing TAPII‐ and Tasso+‐derived capillary plasma biomarker measurements with matched venous plasma measurements, including intercepts, slopes, 95% confidence intervals, and CUSUM test results for neurofilament light chain (NfL), glial fibrillary acidic protein (GFAP), amyloid‐β42/40 (Aβ42/40), and phosphorylated tau 217 (pTau217).

**Table S5:** Age‐adjusted logistic regression models and AUC values for individual biomarkers across venous and capillary plasma sample types. Table presenting age‐adjusted logistic regression model performance and area under the receiver operating characteristic curve (AUC) values with 95% confidence intervals for neurofilament light chain (NfL), glial fibrillary acidic protein (GFAP), amyloid‐β42/40 (Aβ42/40), and phosphorylated tau 217 (pTau217) across venous plasma, TAPII‐derived capillary plasma, and Tasso+‐derived capillary plasma samples.

**Table S6:** Lasso regression models for TAPII, Tasso+, and venous predictors. Table presenting lasso regression model results for TAPII‐derived capillary plasma, Tasso+‐derived capillary plasma, and venous plasma predictors.

**Table S7:** Hemolysis‐adjusted regression models. Table presenting hemolysis‐adjusted regression analyses assessing agreement between TAP‐derived capillary plasma and venous plasma biomarker measurements.

**Table S8:** Associations between capillary plasma biomarkers and cognitive measures. Table reporting correlations between TAPII‐ and Tasso+‐derived capillary plasma neurofilament light chain (NfL) and glial fibrillary acidic protein (GFAP) concentrations and cognitive scores, including the Montreal Cognitive Assessment (MoCA) and Clinical Dementia Rating–Sum of Boxes (CDR‐SOB).

## Data Availability

The raw data that support the findings of this article are available on request to the corresponding author.
