# Supplementary material for: Alzheimer's Disease Biomarkers Measured by Capillary Blood Collection Versus Venous Plasma and Cerebrospinal Fluid
Source: Ann Clin Transl Neurol. 2026 Oct 16:10.1002/acn3.70524. Online ahead of print. doi: 10.1002/acn3.70524 (PMC13674320; doi:10.1002/acn3.70524)
Supplement: Supplementary file 1 [file ACN3-9999-0-s001.docx]

Supplemental Table 1. Sample characteristics

|  | TAPII | Tasso+ | Venous |
| --- | --- | --- | --- |
| Number of samples | **86** | **126** | **169** |
| Volume, mean (SD) | **266.936 ± 118.71 uL** | **294.03 ± 127.29 uL** | **N/a** |
| Pain NRS, mean (SD) | **0.73 ± 1.07** | **0.2 ± 0.40** | **2.24 ± 1.97** |
| Hemolysis, mean (SD) | **2.08 ± 0.75** | **1.37 ± 0.55** | **N/a** |
