# Supplementary material for: Alzheimer's Disease Biomarkers Measured by Capillary Blood Collection Versus Venous Plasma and Cerebrospinal Fluid
Source: Ann Clin Transl Neurol. 2026 Oct 16:10.1002/acn3.70524. Online ahead of print. doi: 10.1002/acn3.70524 (PMC13674320; doi:10.1002/acn3.70524)
Supplement: Supplementary file 2 [file ACN3-9999-0-s002.docx]

Supplemental Table 8. Hemolysis-Adjusted Regression Models Assessing Agreement Between TAP-Derived and Venous Plasma Biomarkers

| **Characteristic** | **Beta** | **95% CI** | p-value |
| --- | --- | --- | --- |
| TAPII NF-light vs Venous NF-light correlation | 0.92 | 0.62, 1.2 | <0.001 |
| Hemolysis Level 1 | — | — |  |
| Hemolysis Level 2 | -4.3 | -15, 6.0 | 0.4 |
| Hemolysis Level 3 | 3.4 | -11, 17 | 0.6 |
| Hemolysis Level 4 | -3.2 | -27, 21 | 0.8 |
| TAPII vs Venous with Hemolysis Level 2 | 0.23 | -0.14, 0.59 | 0.2 |
| TAPII vs Venous with Hemolysis Level 3 | -0.42 | -1.0, 0.15 | 0.15 |
| TAPII vs Venous with Hemolysis Level 4 | -0.01 | -0.94, 0.93 | >0.9 |
|  | **Beta** | **95% CI** | **p-value** |
| TAPII GFAP vs Venous GFAP correlation | 0.74 | 0.34, 1.1 | <0.001 |
| Hemolysis Level 1 | — | — |  |
| Hemolysis Level 2 | 24 | -52, 99 | 0.5 |
| Hemolysis Level 3 | -1.6 | -103, 100 | >0.9 |
| Hemolysis Level 4 | -35 | -156, 86 | 0.6 |
| TAPII vs Venous with Hemolysis Level 2 | -0.22 | -0.67, 0.22 | 0.3 |
| TAPII vs Venous with Hemolysis Level 3 | -0.12 | -0.95, 0.72 | 0.8 |
| TAPII vs Venous with Hemolysis Level 4 | 0.05 | -0.51, 0.60 | 0.9 |
|  | **Beta** | **95% CI** | **p-value** |
| TAPII AB42/40 vs Venous AB42/40 correlation | 0.51 | -0.10, 1.1 | 0.1 |
| Hemolysis Level 1 | — | — |  |
| Hemolysis Level 2 | -0.01 | -0.04, 0.02 | 0.6 |
| Hemolysis Level 3 | -0.02 | -0.06, 0.02 | 0.3 |
| Hemolysis Level 4 | -0.04 | -0.09, 0.01 | 0.1 |
| TAPII vs Venous with Hemolysis Level 2 | 0.17 | -0.46, 0.79 | 0.6 |
| TAPII vs Venous with Hemolysis Level 3 | 0.41 | -0.42, 1.2 | 0.3 |
| TAPII vs Venous with Hemolysis Level 4 | 1 | -0.26, 2.3 | 0.12 |
|  | **Beta** | **95% CI** | **p-value** |
| TAPII pTau217 vs Venous pTau217 correlation | 0.01 | -0.01, 0.03 | 0.2 |
| Hemolysis Level 1 | — | — |  |
| Hemolysis Level 2 | 0.09 | -0.10, 0.28 | 0.3 |
| Hemolysis Level 3 | -0.01 | -0.30, 0.29 | >0.9 |
| Hemolysis Level 4 | -0.26 | -0.57, 0.05 | 0.1 |
| TAPII vs Venous with Hemolysis Level 2 | -0.01 | -0.02, 0.01 | 0.5 |
| TAPII vs Venous with Hemolysis Level 3 | -0.01 | -0.04, 0.02 | 0.5 |
| TAPII vs Venous with Hemolysis Level 4 | 0.06 | 0.03, 0.09 | <0.001 |
|  | **Beta** | **95% CI** | **p-value** |
| Tasso+ NF-light vs Venous NF-light correlation | 0.84 | 0.70, 0.97 | <0.001 |
| Hemolysis Level 2 | — | — |  |
| Hemolysis Level 3 | -4.3 | -15, 6.6 | 0.4 |
| Hemolysis Level 4 | -10 | -41, 21 | 0.5 |
| Tasso+ vs Venous with Hemolysis Level 3 | 0.07 | -0.35, 0.49 | 0.7 |
| Tasso+ vs Venous with Hemolysis Level 4 | 0.36 | -0.64, 1.3 | 0.5 |
|  | **Beta** | **95% CI** | **p-value** |
| Tasso+ GFAP vs Venous GFAP correlation | 0.76 | 0.68, 0.85 | <0.001 |
| Hemolysis Level 2 | — | — |  |
| Hemolysis Level 3 | -42 | -75, -9.2 | 0.013 |
| Hemolysis Level 4 | -75 | -262, 113 | 0.4 |
| Tasso+ vs Venous with Hemolysis Level 3 | 0.27 | 0.08, 0.46 | 0.006 |
| Tasso+ vs Venous with Hemolysis Level 4 | 0.56 | -0.79, 1.9 | 0.4 |
|  | **Beta** | **95% CI** | **p-value** |
| Tasso+ AB42/40 vs Venous AB42/40 correlation | 0.93 | 0.80, 1.1 | <0.001 |
| Hemolysis Level 2 | — | — |  |
| Hemolysis Level 3 | -0.01 | -0.02, 0.01 | 0.5 |
| Hemolysis Level 4 | 0 | -0.04, 0.05 | >0.9 |
| Tasso+ vs Venous with Hemolysis Level 3 | 0.18 | -0.14, 0.50 | 0.3 |
| Tasso+ vs Venous with Hemolysis Level 4 | -0.04 | -0.99, 0.91 | >0.9 |
|  | **Beta** | **95% CI** | **p-value** |
| Tasso+ pTau217 vs Venous pTau217 correlation | 0.04 | 0.02, 0.07 | 0.002 |
| Hemolysis Level 2 | — | — |  |
| Hemolysis Level 3 | 0.15 | -0.08, 0.38 | 0.2 |
| Hemolysis Level 4 | -0.26 | -1.1, 0.58 | 0.5 |
| Tasso+ vs Venous with Hemolysis Level 3 | -0.05 | -0.11, 0.01 | 0.091 |
| Tasso+ vs Venous with Hemolysis Level 4 | 0.14 | -0.44, 0.71 | 0.6 |
