# Supplementary material for: Alzheimer's Disease Biomarkers Measured by Capillary Blood Collection Versus Venous Plasma and Cerebrospinal Fluid
Source: Ann Clin Transl Neurol. 2026 Oct 16:10.1002/acn3.70524. Online ahead of print. doi: 10.1002/acn3.70524 (PMC13674320; doi:10.1002/acn3.70524)
Supplement: Supplementary file 3 [file ACN3-9999-0-s004.docx]

Supplementary Table 4. Passing-Bablok Regression Statistics for Agreement Between Capillary and Venous Plasma Biomarkers

| Capillary Device | Biomarker | Intercept (95% CI) | Slope (95% CI) |
| --- | --- | --- | --- |
| TAP | NFlight | -0.023 (-1.150, 0.843) | 0.975 (0.893, 1.081) |
| TASSO | NFlight | -0.459 (-1.313, 0.925) | 0.985 (0.910, 1.063) |
| TAP | GFAP | -0.118 (-8.146, 7.955) | 1.127 (0.992, 1.244) |
| TASSO | GFAP | 4.853 (-2.720, 11.236) | 0.990 (0.900, 1.071) |
| TAP | AB4240 | 0.004 (-0.004, 0.010) | 0.934 (0.793, 1.122) |
| TASSO | AB4240 | 0.006 (0.001, 0.010) | 0.843 (0.748, 0.950) |
| TAP | pTau217 | -22.3 (-184.2, -8.7) | 218.9 (109.2, 1351.3) |
| TASSO | pTau217 | -2.3 (-5.8, -0.6) | 25.4 (14.9, 41.8) |
