# Supplementary material for: Alzheimer's Disease Biomarkers Measured by Capillary Blood Collection Versus Venous Plasma and Cerebrospinal Fluid
Source: Ann Clin Transl Neurol. 2026 Oct 16:10.1002/acn3.70524. Online ahead of print. doi: 10.1002/acn3.70524 (PMC13674320; doi:10.1002/acn3.70524)
Supplement: Supplementary file 4 [file ACN3-9999-0-s005.docx]

Supplemental Table 7. Correlations between TAPII and Tasso+ Nfl and GFAP and MoCA/CDR-SOB

| **Capillary Device** | **Cognitive Test** | **Analyte** | **Correlation Test** | **Estimated rho** | **P value** | **Transformation** |
| --- | --- | --- | --- | --- | --- | --- |
| TAPII | MoCA | NF-light | Pearson | -0.27 | 0.1 | N/a |
|  |  | GFAP | Pearson | -0.47 | 0.003154 | log(GFAP) |
| TAPII | CDR-SOB | NF-light | Spearman | -0.00273 | 0.99 | N/a |
|  |  | GFAP | Spearman | 0.16 | 0.368 | N/a |
| Tasso+ | MoCA | NF-light | Spearman | -0.09 | 0.44 | N/a |
|  |  | GFAP | Spearman | -0.18 | 0.14 | N/a |
| Tasso+ | CDR-SOB | NF-light | Spearman | 0.23 | 0.067 | N/a |
|  |  | GFAP | Spearman | 0.17 | 0.19 | N/a |
