# Supplementary material for: Alzheimer's Disease Biomarkers Measured by Capillary Blood Collection Versus Venous Plasma and Cerebrospinal Fluid
Source: Ann Clin Transl Neurol. 2026 Oct 16:10.1002/acn3.70524. Online ahead of print. doi: 10.1002/acn3.70524 (PMC13674320; doi:10.1002/acn3.70524)
Supplement: Supplementary file 5 [file ACN3-9999-0-s009.docx]

Supplemental Table 3. Intraclass Correlation Coefficients and Bland-Altman Metrics for Agreement Between Capillary and Venous Plasma Biomarkers

| Device | Analyte | ICC | F | p value | lower bound | upper bound |
| --- | --- | --- | --- | --- | --- | --- |
| TAPII | NfL | 0.84 | 12 | 7.30E-21 | 0.76 | 0.9 |
|  | GFAP | 0.69 | 5.5 | 7.60E-12 | 0.55 | 0.79 |
|  | AB42/40 | 0.74 | 6.8 | 2.10E-14 | 0.62 | 0.83 |
|  | ptau217 | -0.4252 | 0.4 | 1.00E+00 | -0.59 | -0.22 |
| Tasso+ | NfL | 0.8 | 8.9 | 6.90E-25 | 0.72 | 0.86 |
|  | GFAP | 0.89 | 18 | 4.20E-38 | 0.85 | 0.93 |
|  | AB42/40 | 0.84 | 12 | 3.90E-30 | 0.78 | 0.89 |
|  | ptau217 | -0.369 | 0.46 | 1.00E+00 | -0.522 | -0.19 |
