# Supplementary material for: Alzheimer's Disease Biomarkers Measured by Capillary Blood Collection Versus Venous Plasma and Cerebrospinal Fluid
Source: Ann Clin Transl Neurol. 2026 Oct 16:10.1002/acn3.70524. Online ahead of print. doi: 10.1002/acn3.70524 (PMC13674320; doi:10.1002/acn3.70524)
Supplement: Supplementary file 6 [file ACN3-9999-0-s011.docx]

Supplemental Table 6. Lasso Regression Models for TAPII, Tasso+, and Venous predictors

| **TAPII** |  | **Tasso+** |  | **Venous** |  |
| --- | --- | --- | --- | --- | --- |
| **Predictor** | **Coefficient** | **Predictor** | **Coefficient** | **Predictor** | **Coefficient** |
| (Intercept) | -7.644205419 | (Intercept) | -4.457294677 | (Intercept) | -4.999130919 |
| Age | 0.075058384 | Age | 0.025123888 | Age | 0.034490097 |
| AB42/40 | -3.927646831 | GFAP | 0.005794278 | GFAP | 0.005020479 |
| GFAP | 0.004387818 |  |  |  |  |
|  |  |  |  |  |  |
| **AUC** | **0.7546** | **AUC** | **0.8732582** | **AUC** | **0.8384972** |
