# Supplementary material for: Alzheimer's Disease Biomarkers Measured by Capillary Blood Collection Versus Venous Plasma and Cerebrospinal Fluid
Source: Ann Clin Transl Neurol. 2026 Oct 16:10.1002/acn3.70524. Online ahead of print. doi: 10.1002/acn3.70524 (PMC13674320; doi:10.1002/acn3.70524)
Supplement: Supplementary file 7 [file ACN3-9999-0-s013.docx]

Supplementary Table 5. Age-adjusted logistic regression models and AUC values for individual biomarkers across venous and capillary plasma sample types.

| **Predictor** | **Venous** **(N=164*)** | **TAPII** **(N = 85**)** | **Tasso+** **(N = 121***)** | **P value** |
| --- | --- | --- | --- | --- |
| NfL | 0.79 (0.71, 0.88) | 0.84 (0.72, 0.95) | 0.78 (0.68, 0.88) | 0.77 |
| GFAP | 0.85 (0.78, 0.92) | 0.88 (0.80, 0.97) | 0.82 (0.73, 0.90) | 0.59 |
| AB42/40 | 0.78 (0.70, 0.87) | 0.86 (0.76, 0.96) | 0.82 (0.72, 0.91) | 0.50 |
| ptau217 | 0.88 (0.82, 0.94) | 0.84 (0.73, 0.95) | 0.79 (0.70, 0.89) | 0.34 |
