# Supplementary material for: Alzheimer's Disease Biomarkers Measured by Capillary Blood Collection Versus Venous Plasma and Cerebrospinal Fluid
Source: Ann Clin Transl Neurol. 2026 Oct 16:10.1002/acn3.70524. Online ahead of print. doi: 10.1002/acn3.70524 (PMC13674320; doi:10.1002/acn3.70524)
Supplement: Supplementary file 8 [file ACN3-9999-0-s015.docx]

Supplemental Table 2. Mean concentrations and standard deviation of Nfl, GFAP, Aβ42/40, and pTau217 in TAPII, Tasso+. Venous plasma, and CSF samples

| Sample Type | Analyte | AD | IIH | NPH | ON | Other | VaD | YN |
| --- | --- | --- | --- | --- | --- | --- | --- | --- |
| CSF | Aβ42/40 | 0.049±0.014 | 0.088±0.007 | 0.084±0.015 | 0.085±0.017 | 0.068±0.02 | 0.083±0.02 | 0.09±0.008 |
| TAPII | Aβ42/40 | 0.037±0.007 | 0.045±0.011 | 0.045±0.009 | 0.048±0.007 | 0.037±0.005 | 0.043±0.01 | 0.053±0.013 |
| TASSO+ | Aβ42/40 | 0.04±0.012 | 0.045±0.011 | 0.044±0.011 | 0.045±0.017 | 0.055±0.026 | 0.05±0.011 | 0.051±0.008 |
| Venous | Aβ42/40 | 0.042±0.011 | 0.045±0.013 | 0.045±0.013 | 0.042±0.015 | 0.047±0.023 | 0.045±0.013 | 0.05±0.012 |
| CSF | GFAP | 45616.06±34430.52 | 11728.5±14960.19 | 26577.64±14784.98 | 18847.65±8710.81 | 28230.04±6817.78 | 26844.69±20294.36 | 9028.75±4808.78 |
| TAPII | GFAP | 245.514±120.022 | 85.26±98.361 | 136.839±95.107 | 109.137±61.184 | 188.246±72.171 | 166.7±86.934 | 61.99±21.554 |
| TASSO+ | GFAP | 246.471±158.923 | 63.021±20.945 | 156.123±98.283 | 144.954±57.554 | 210.091±133.390 | 126.801±68.581 | 81.120±60.565 |
| Venous | GFAP | 235.910±109.620 | 58.951±32.804 | 145.177±96.910 | 111.481±35.310 | 228.090±118.286 | 128.612±69.890 | 69.021±49.981 |
| CSF | Nfl | 4330.27±5496.69 | 990.69±1330.89 | 2256.61±1430.63 | 1726.32±1182.35 | 3577.29±3098.49 | 2247.52±1371.79 | 944.67±1109.43 |
| TAPII | Nfl | 28.493±12.681 | 11.164±10.384 | 28.274±14.985 | 19.883±11.848 | 32.903±23.11 | 28.32±9.587 | 9.606±8.607 |
| TASSO+ | Nfl | 40.172±31.432 | 10.360±6.851 | 28.840±18.791 | 21.950±11.391 | 35.015±19.781 | 25.22±10.57 | 8.89±5.65 |
| Venous | Nfl | 41.844±29.280 | 11.321±14.850 | 30.844±19.942 | 20.311±9.082 | 43.223±42.301 | 26.080±12.150 | 11.082±7.472 |
| CSF | pTau217 | 64.56±15.013 | 12.89±2.991 | 12.1±2.813 | 10.94±2.544 | 29.15±6.779 | 16.5±3.837 | 9.25±2.151 |
| TAPII | pTau217 | 12.482±10.562 | 6.627±4.853 | 10.059±6.075 | 9.189±5.668 | 14.866±15.189 | 7.491±4.955 | 6.940±4.019 |
| TASSO+ | pTau217 | 4.310±2.660 | 2.661±1.450 | 3.060±2.133 | 4.240±2.678 | 3.580±2.231 | 3.001±2.450 | 4.344±4.244 |
| Venous | pTau217 | 0.683±0.321 | 0.131±0.050 | 0.282±0.164 | 0.210±0.249 | 0.460±0.331 | 0.350±0.362 | 0.180±0.221 |
